# Ensuring expression of four core cardiogenic transcription factors enhances cardiac reprogramming

**DOI:** 10.1038/s41598-019-42945-w

**Published:** 2019-04-24

**Authors:** Zhentao Zhang, Alexander D. Zhang, Luke J. Kim, Young-Jae Nam

**Affiliations:** 1 Department of Medicine, Division of Cardiovascular Medicine, Vanderbilt University Medical Center, Nashville, TN USA; 2 Department of Cell and Developmental Biology, Vanderbilt University, Nashville, TN USA; 3 Vanderbilt Center for Stem Cell Biology, Vanderbilt University, Nashville, TN USA

**Keywords:** Reprogramming, Cardiac regeneration

## Abstract

Previous studies have shown that forced expression of core cardiogenic transcription factors can directly reprogram fibroblasts to induced cardiomyocyte-like cells (iCMs). This cardiac reprogramming approach suggests a potential strategy for cardiomyocyte regeneration. However, a major challenge of this approach remains the low conversion rate. Here, we showed that ensuring expression of four cardiogenic transcription factors (i.e. Gata4 (G), Hand2 (H), Mef2c (M), and Tbx5 (T)) in individual fibroblasts is an initial bottleneck for cardiac reprogramming. Following co-transduction of three or four retroviral vectors encoding individual cardiogenic transcription factors, only a minor subpopulation of cells indeed expressed all three (GMT) or four (GHMT) factors. By selectively analyzing subpopulations of cells expressing various combinations of reprogramming factors, we found that co-expression of GMT in individual fibroblasts is sufficient to induce sarcomeric proteins. However, only a small fraction of those cells expressing GMT were able to develop organized sarcomeric structures and contractility. In contrast, ensuring expression of GHMT markedly enhanced the development of contractile cardiac structures and functions in fibroblasts, although its incremental effect on sarcomeric protein induction was relatively small. Our findings provide new insights into the mechanistic basis of inefficient cardiac reprogramming and can help to devise efficient reprogramming strategies.

## Introduction

The discovery of induced pluripotent stem cells (iPSCs) has established the concept of cell fate reprogramming by demonstrating that ectopic expression of lineage specific transcription factors can convert one differentiated cell type to another^1^. In fact, this concept of cell fate reprogramming has long been known from earlier studies, but was limited to cell types where a single master transcription factor was identified^2–5^. However, the discovery of iPSCs demonstrated a previously unrecognized requirement of multiple transcription factors, rather than a single factor, for cell fate reprogramming. Based on this advanced concept of cell fate reprogramming using multiple transcription factors, Srivastava’s group first reported conversion of fibroblasts to induced cardiomyocyte-like cells (iCMs) by forced expression of three core cardiogenic transcription factors (i.e. Gata4 (G), Mef2c (M), and Tbx5 (T); referred to as GMT)^6^. The subsequent study by Olson’s group further optimized cardiac reprogramming by adding Hand2 (H) into GMT combination (referred to as GHMT)^7^. Importantly, several groups demonstrated direct reprogramming of resident cardiac fibroblasts in the heart to iCMs following cardiac injury by forced expression of GMT or GHMT *in vivo*^7–12^. Taken together, cardiac reprogramming may provide a potential platform to develop therapeutic strategies for heart diseases and *in vitro* clinical applications for drug screening or heart disease modeling.

One major hurdle for realizing the attractive potential applications of cardiac reprogramming is the low conversion rate of fibroblasts to iCMs. Numerous approaches have been tested to enhance cardiac reprogramming efficiency, mainly by adding additional genetic factors or small molecules. For example, adding microRNA-133^13,14^, microRNA-1^14^, Bmi1^15^, Akt1^16^, or Znf281^17^ into GMT or GHMT has been shown to increase cardiac reprogramming efficiency. In addition, pharmacological manipulations of Tgf-β^14,18^, Wnt^11^, Notch^19^, p38 mitogen activated protein kinase and phosphoinositol 3-kinase pathways^20^ have shown to enhance cardiac reprogramming. However, a significant population of transduced cells still remain unreprogrammed, suggesting fundamental differences between reprogrammed and unreprogrammed cell populations following transduction of viral vectors encoding reprogramming factors. That made us speculate that the effects of additional genetic or pharmacological factors may be confined to the selected subpopulation of cells which already passed through an unrecognized upfront bottleneck of cardiac reprogramming. This may explain the limited effects of optimized reprogramming protocols, which enhance the activation of cardiogenic transcriptional networks or regulatory pathways.

In this study, we examined an initial step in the reprogramming process by carefully assessing the exogenous expression profiles of individual reprogramming factors in fibroblasts following transduction. Only a small subpopulation of cells co-expressed all reprogramming factors intended to be overexpressed, suggesting an initial mechanistic cause for low reprogramming efficiency. Through high content imaging analyses of individual subpopulations defined by distinct expression profiles of reprogramming factors, we found that a majority of cells expressing GMT or GHMT were able to induce sarcomeric proteins. Although its incremental effect on sarcomeric protein induction is relatively small, ensuring expression of GHMT markedly enhanced the development of contractile structures and functions in fibroblasts over that of GMT. Taken together, our results identified an initial bottleneck of cardiac reprogramming, and demonstrated the irrefutable effects of Hand2 in the context of GMT expression on cardiac reprogramming.

## Results and Discussion

### Low co-expression efficiency following simultaneous transduction of multiple reprogramming factors

**Figure 1:**
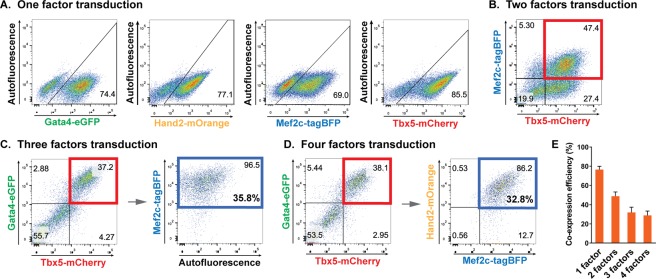
Co-expression efficiency following simultaneous transducing different numbers of retroviral vectors encoding individual reprogramming factors tagged with a distinct fluorescent reporter. MEFs were transduced with indicated vectors. Expression of reprogramming factors was analyzed by expression of tagged fluorescent reporters using flow cytometry. (**A**) Representative flow cytometry analysis showing expression efficiency following a single vector transduction. The number indicates the percentage of cells expressing a single factor following single vector transduction. (**B**) Representative flow cytometry analysis showing co-expression efficiency for two factors following simultaneous transduction of two vectors. The number in the red box indicates the percentage of cells expressing both factors. (**C**) Representative flow cytometry analysis showing co-expression efficiency for three factors following simultaneous transduction of three vectors. The number in the red box indicates the percentage of cells expressing two factors following transduction of three vectors. The top number in the blue box indicates the percentage of cells expressing the third factor among cells expressing two factors indicated by red box. The bottom number in the blue box indicates the percentage of cells expressing all three factors in the whole cell population (37.2 × 0.965 = 35.8%). (**D**) Representative flow cytometry analysis showing co-expression efficiency for four factors following simultaneous transduction of four vectors. The number in the red box indicates the percentage of cells expressing two factors following transduction of four vectors. The top number in the blue box indicates the percentage of cells expressing the other two factors among the cellular population expressing the two factors indicated by red box. The bottom number in the blue box indicates the percentage of cells expressing all four factors in the whole cell population (38.1 × 0.862 = 32.8%). (**E**) A summary graph of co-expressing efficiency for indicated number(s) of factors. Data from three or four independent experiments are presented as means ± s.d.

Previous studies assessed the reprogramming efficiency of whole cell populations following the transduction of multiple viral vectors harboring individual reprogramming factors, assuming that most of transduced cells uniformly expressed all factors. We hypothesized that low cardiac reprogramming efficiency is, at least in part, due to incomplete expression of the whole set of defined reprogramming factors (GMT or GHMT) in fibroblasts. To test this hypothesis, we first generated retroviral constructs harboring individual reprogramming factors tagged with four different fluorescent reporters (i.e. Gata4-eGFP, Hand2-mOrange, Mef2c-tagBFP, and Tbx5-mCherry). We transduced one, two, three, or four retroviral vectors encoding individual reprogramming factors into mouse embryonic fibroblasts (MEFs). Four days later, we analyzed the transduced MEFs using flow cytometry to quantify the fraction of cells expressing the various numbers of reprogramming factors (Fig. 1). Single vector transduction resulted in expression of a fluorescent reporter harbored in an individual vector in ~70–85% of cells (Fig. 1A). However, we found that the number of cells expressing all the transduced factors significantly decreased when multiple vectors were co-transduced (Fig. 1B–E). Transduction of two vectors showed ~50% co-expression efficiency assessed by quantifying the percentage of cells expressing both fluorescent reporters (Fig. 1B). Only a minor fraction of fibroblasts (less than 40%) co-expressed all three or four factors (Fig. 1C–E). Taken together, our results showed that a large fraction of the whole cell population fails to co-express all the reprogramming factors following transduction of multiple viral vectors, each of which encodes a reprogramming factor. These findings indicate that incomplete co-expression of a defined set of reprogramming factors can be a major cause of reprogramming failure in a significant population of cells at the very early step of cardiac reprogramming processes.

### Inefficient cardiac reprogramming by forced expression of two core cardiogenic transcription factors

**Figure 2:**
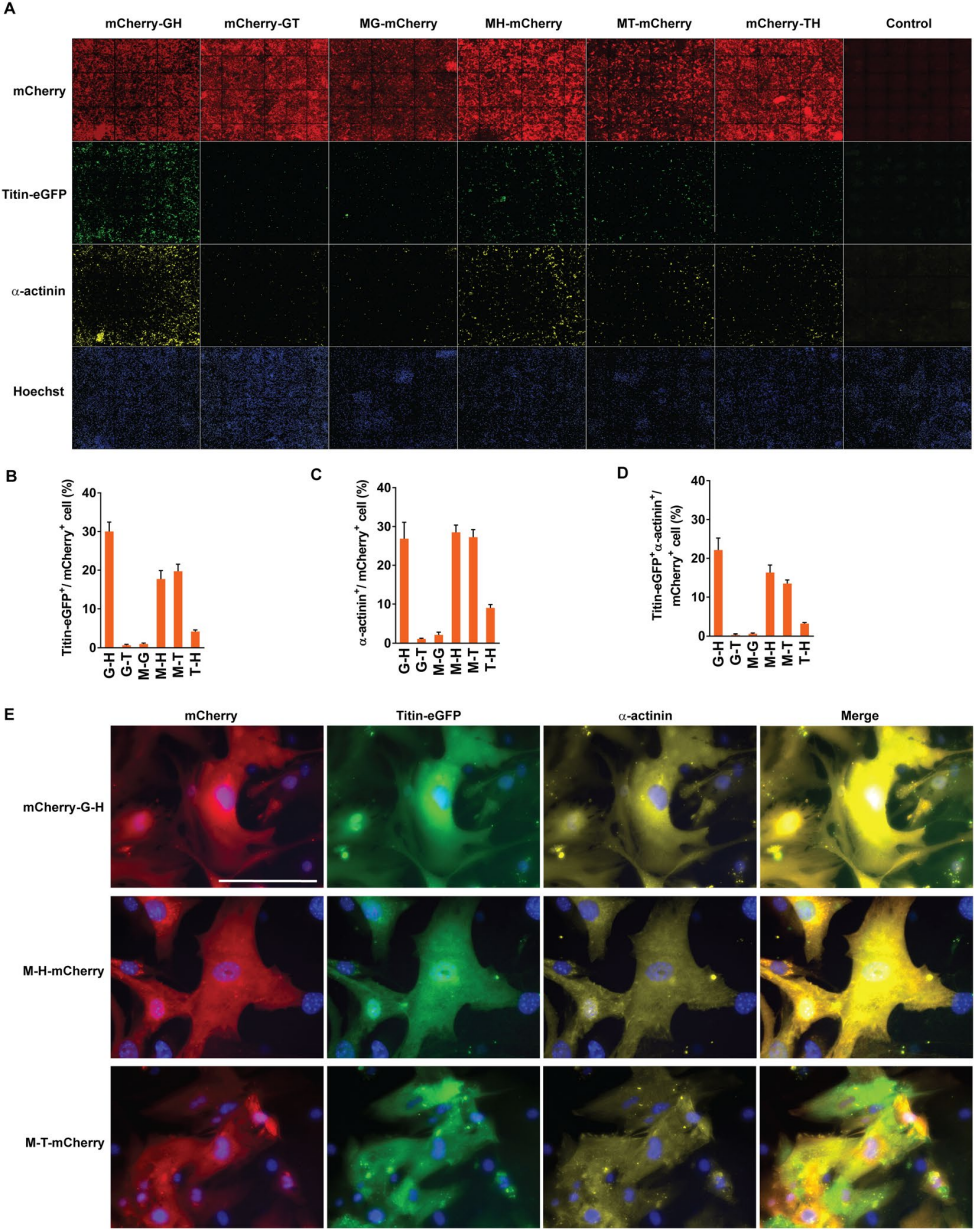
Cardiac reprogramming by combinations of two cardiogenic transcription factors. (**A**) Representative immunofluorescent images used for high content imaging analyses to quantify Titin-eGFP and/or α-actinin expression following transduction of tri-cistronic vectors harboring various combinations of two cardiogenic transcription factors and mCherry. The indicated vectors were transduced into MEFs isolated from Titin-eGFP reporter knock-in mice. Immunofluorescence staining followed by high content imaging analyses was performed at day 15 after transduction. Each panel shows a composition of 36 images taken by 10X objective in the high content imaging system. (**B**–**D**) Summary of high content imaging analyses for the percentage of Titin-eGFP^+^ (**B**), α-actinin^+^ (**C**), or Titin-eGFP^+^ and α-actinin^+^ (**D**) cells among cells expressing various combinations of two reprogramming factors. Three independent experiments are presented as mean ± s.d. (**E**) Representative high magnification views of immunofluorescent images of the cells analyzed by a high content imaging system. Sarcomere protein induction in mCherry-G-H, M-H-mCherry, and M-T-mCherry expressing cells was shown. Scale bar, 100 µM.

Co-expression analysis using a fluorescent reporter-tagged reprograming factors demonstrated that GMT or GHMT transduction can produce a mixture of the cells expressing various combinations of reprogramming factors (i.e. GHMT, GHT, GMT, HMT, GH, GM, GT, HM, HT, G, H, M, and T) (Fig. 1). Previous studies estimated the reprogramming efficiency of whole cell populations by summing the number of reprogrammed cells in each subpopulation expressing a different combination of factors. Therefore, it is still unclear which minimum collection of core cardiogenic transcription factors can induce a cardiac phenotype in individual fibroblasts. We sought to determine whether fibroblasts expressing two reprogramming factors are able to induce a cardiac phenotype. To visualize the cells expressing different combinations of two reprogramming factors, we generated tri-cistronic vectors encoding two reprogramming factors and mCherry (a red fluorescent protein), in which each factor and mCherry are sequentially linked with the self-processing P2A peptide (i.e. mCherry-G-H, mCherry-G-T, M-G-mCherry, M-H-mCherry, M-T-mCherry, and mCherry-T-H) (Supplementary Fig. 1). Using a high content imaging system (ImageXpress Micro High-Content Imaging Systems, Molecular Devices), we analyzed the expression of mCherry and a sarcomeric protein (α-actinin or Titin-eGFP) at day 15 after transducing the tri-cistronic vectors into MEFs isolated from wild type or Titin-eGFP reporter knock-in mice^21^ (Fig. 2). We added TGF-β inhibitors to cardiomyocyte induction media in order to enhance cardiac reprogramming efficiency throughout this study^14,18^. We quantified the percentage of cells expressing a sarcomeric protein among mCherry^+^ cells, representing expression of two reprogramming factors. We found that ~20–30% of GH, MH, or MT expressing fibroblasts induced Titin-eGFP or α-actinin, while only a very small fraction of GT, MG, or TH expressing cells were able to do so (Fig. 2B,C). The percentage of cells expressing both Titin-eGFP and α-actinin among cells expressing two factors was lower than the percentage of cells inducing either sarcomeric protein (Fig. 2D). Given that an organized sarcomeric structure is a hallmark of functional iCMs^22^, we further analyzed the development of organized sarcomeric structures by examining mCherry^+^Titin-eGFP^+^ cells under an epifluorescent microscope (Fig. 2E). Very few mCherry^+^ cells exhibited organized myofibrillar structures (Supplementary Fig. 2), while nearly all mCherry^+^Titin-eGFP^+^ or mCherry^+^α-actinin^+^ cells show diffuse cellular expression patterns of Titin-eGFP or α-actinin without any distinctive cytoskeletal structures (Fig. 2E). These results indicate that the combinations of two factors are not sufficient for sarcomere assembly. Accordingly, there were no spontaneously beating cells observed in mCherry^+^ cellular population. Our results showed that some, but not all, combinations of two reprogramming factors (i.e. GH, MH, and MT) are able to induce sarcomeric proteins in a significant number of cells expressing the two factors, but ultimately fail to develop organized sarcomeric structures. These findings indicate that any combination of two factors is not sufficient for developing contractile cardiac structures and functions in fibroblasts.

### Efficient sarcomeric protein induction in the subpopulation of cells expressing a complete set of defined factors

**Figure 3:**
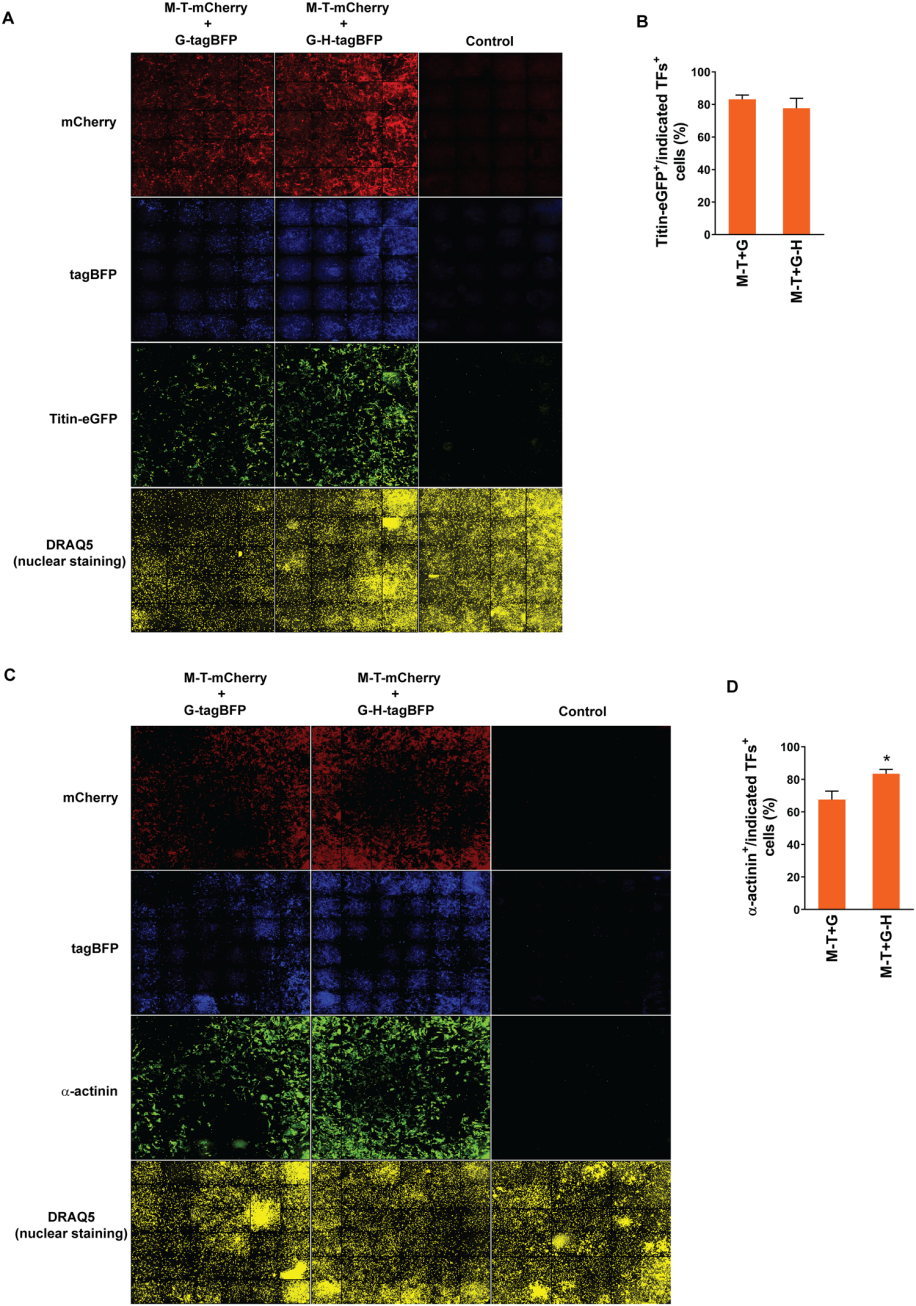
Quantification of sarcomeric protein induction in GMT or GHMT expressing fibroblasts by high content imaging analyses. (**A**) Representative immunofluorescent images used for high content imaging analyses to quantify Titin-eGFP expression following M-T-mCherry and G-tagBFP or G-H-tagBFP transduction. The indicated combinations of vectors were transduced into MEFs isolated from Titin-eGFP reporter knock-in mice. Immunofluorescence staining followed by high content imaging analyses was performed at day 15 post-transduction. Each panel shows a composition of 20 images taken by the 10X objective in the high content imaging system. (**B**) Summary of high content imaging analyses for the percentage of Titin-eGFP^+^ cells among cells expressing GMT or GHMT. Six independent experiments are presented as mean ± s.d. *P* > 0.5. (**C**) Representative immunofluorescent images used for high content imaging analyses to quantify α-actinin expression following M-T-mCherry and G-tagBFP or G-H-tagBFP transduction into MEFs. The indicated combinations of vectors were transduced into MEFs isolated from wild type mice. Immunofluorescence staining followed by high content imaging analyses was performed at day 15 post-transduction. Each panel shows a composition of 36 images taken by the 10X objective in the high content imaging system. (**D**) Summary of high content imaging analyses for the percentage of α-actinin^+^ cells among cells expressing GMT or GHMT. Six independent experiments are presented as mean ± s.d. *P < 0.05.

Given the poor co-expression profiles of reprogramming factors by co-transduction of individual viral vectors and the low reprogramming efficiency and quality in selected cellular populations expressing two factors, we hypothesized that ensuring expression of all three (GMT) or four (GHMT) defined factors in individual fibroblasts will enhance reprogramming efficiency and quality. All the previous cardiac reprogramming studies estimated reprogramming efficiency by quantifying the fraction of cells exhibiting cardiac phenotypes amongst the whole cell population. Therefore, it is unknown how efficiently the fibroblasts expressing all three or four reprogramming factors are converted into iCMs. To determine the reprogramming efficiency in the fibroblast population co-expressing GMT or GHMT, we generated bi- and tri-cistronic vectors encoding Gata4 or Gata4 and Hand2 along with tagBFP (a blue fluorescent protein), each of which is linked by the self-processing P2A peptide (i.e. G-tagBFP and G-H-tagBFP), in addition to M-T-mCherry described above (Supplementary Fig. 3). After transducing two of these vectors together (i.e. M-T-mCherry plus G-tagBFP and M-T-mCherry plus G-H-tagBFP), we were able to identify cells expressing GMT or GHMT by demonstrating both mCherry and tagBFP fluorescence. Using a high content imaging system, we quantified the percentage of cells expressing a sarcomeric protein (Titin-eGFP or α-actinin) among cells expressing both fluorescent reporters which represent the cellular population expressing all three or four reprogramming factors (Fig. 3A–D, and Supplementary Fig. 4). We found that ~70–80% of cells expressing all three or four factors induced Titin-eGFP or α-actinin expression in fibroblasts. There was no statistically significant difference in Titin-eGFP induction between subpopulations of cells expressing GMT and GHMT (Fig. 3B). However, there was a relatively small, but statistically significant, enhancement of α-actinin induction in GHMT expressing cells over GMT expressing cells (Fig. 3D). Furthermore, we assessed induction of ventricular specific sarcomeric protein, MLC-2v, in GMT or GHMT expressing cells. We transduced M-T-eGFP and G-tagBFP or G-H-tagBFP into MEFs isolated from MLC-2v-tdTomato reporter knock-in mice in which tdTomato expression precisely recapitulates endogenous MLC-2v expression^23^. In contrast to Titin or α-actinin protein induction, ventricular specific MLC-2v induction was very inefficient in both GMT and GHMT expressing fibroblasts (Supplementary Fig. 5). Consistent with our previous study, this result suggests that subtype specification of directly reprogrammed iCMs is incomplete^22^, and requires more than forced expression of GMT or GHMT in individual fibroblasts. Taken together, our results demonstrated that co-expression of GMT or GHMT in fibroblasts is sufficient to induce sarcomeric proteins involved in contractile structures of a cardiomyocyte regardless of a cardiac subtype. This efficiency for sarcomeric protein induction in the selected population expressing GMT or GHMT is much higher than the one estimated in the whole cell population.

### Development of contractile structures and functions in fibroblasts is enhanced by ensuring expression of four core cardiogenic transcription factors

**Figure 4:**
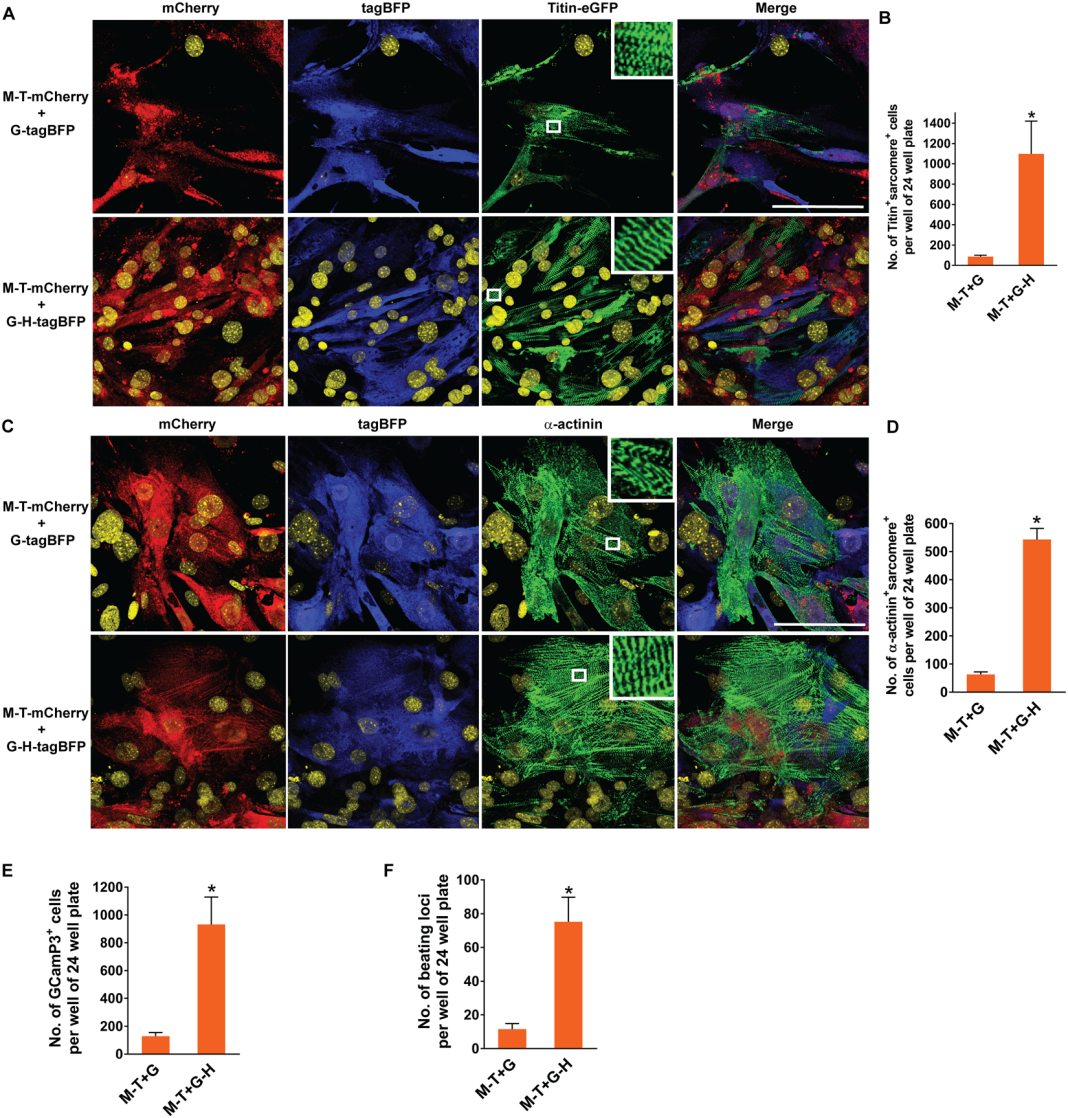
Structural and functional quality of the iCMs derived from GMT or GHMT expressing fibroblasts. (**A**) Immunofluorescence staining of M-T-mCherry and G-tagBFP or G-H-tagBFP transduced Titin-eGFP reporter MEFs for mCherry (red), tagBFP (blue), and Titin-eGFP (green). The indicated combinations of vectors were transduced into MEFs isolated from Titin-eGFP reporter knock-in mice. Immunofluorescence staining was performed at day 15 post-transduction. White boxes are enlarged in insets to demonstrate M-band structures in the sarcomere. Nuclei are stained with DRAQ5 (yellow). Scale bar, 100 µM. (**B**) Quantification of well-organized sarcomere^+^ cells identified by visualizing M-band structures with Titin-eGFP expression after M-T-mCherry and G-tagBFP or G-H-tagBFP transduction. Well-organized sarcomeric structures were counted on 40X fields of epifluorescent microscope. Four independent experiments are presented as mean ± s.d. *P < 0.05. (**C**) Immunofluorescence staining of M-T-mCherry and G-tagBFP or G-H-tagBFP transduced wild type MEFs for mCherry (red), tagBFP (blue), and α-actinin (green). Immunofluorescence staining was performed at day 15 post-transduction. White boxes are enlarged in insets to demonstrate Z-band structures in the sarcomere. Scale bar, 100 µM. (**D**) Quantification of well-organized sarcomere^+^ cells identified by visualizing Z-band structures with α-actinin expression after M-T-mCherry and G-tagBFP or G-H-tagBFP transduction. Four independent experiments are presented as mean ± s.d. *P < 0.0001. (**E**) Quantification of GCaMP3^+^ cells following M-T-mCherry and G-tagBFP or G-H-tagBFP transduction into MEFs isolated from αMHC-Cre: Rosa26-GCaMP3 mice. Calcium oscillation identified by flashing green fluorescence was counted on 20X fields of epifluorescent microscope at day 20 or 21 post-transduction. Eight independent experiments are presented as mean ± s.d. *P < 0.005. (**F**) Quantification of spontaneously beating loci following M-T-mCherry and G-tagBFP or G-H-tagBFP transduction. Beating loci were counted at day 18 or D19 post- transduction. Four independent experiments are presented as mean ± s.d. *P < 0.01.

Previous studies have estimated cardiac reprogramming efficiency by assessing induction of sarcomeric proteins (e.g. cTnT, α-actinin, and αMHC)^6,7,13,14,16,17,24–27^. Although necessary, sarcomeric protein induction in fibroblasts alone is not sufficient for generating functional iCMs. We previously showed that the development of well-organized sarcomeric structures is required for contractile function of iCMs^22^. Thus, we further analyzed the development of organized sarcomeric structures following transduction of M-T-mCherry plus G-tagBFP or M-T-mCherry plus G-H-tagBFP. Organized sarcomeric assembly was evaluated by demonstrating M-band and Z-band structures by Titin-eGFP and α-actinin immunostaining, respectively. Because eGFP was inserted into M-band exon 6 of Titin in Titin-eGFP reporter knock-in mice, Titin-eGFP knock-in reporter expression demonstrates M-band in the sarcomere^21^. A significantly greater number of cells expressing GHMT demonstrated well-organized sarcomeric structures detected by both Titin-eGFP and α-actinin immunostaining over cells expressing GMT (Fig. 4A–D). Next, we examined functionality of the cells reprogrammed with GMT or GHMT by assessing their spontaneous calcium oscillation and contraction. To evaluate calcium oscillation, we used MEFs isolated from the mice carrying αMHC-Cre and GCaMP3 alleles, in which activation of cardiac specific αMHC promoter induces expression of a calcium reporter, GCaMP3^16^. A significantly greater number of GHMT expressing cells revealed spontaneous calcium oscillation by GCaMP3 over GMT expressing cells (Fig. 4E and Supplementary Movies 1–4). Similarly, about an eight-fold greater number of cells expressing GHMT demonstrated spontaneous contraction over GMT expressing cells (Fig. 4F). Using Titin-eGFP reporter MEFs, we simultaneously visualized both organized-sarcomeric structures and their spontaneous contraction in GMT or GHMT expressing live cells (Supplementary Movies 5–8 and Fig. 6). Taken together, our results demonstrated the importance of ensuring expression of four core cardiogenic transcription factors (GHMT) for the development of sarcomeric organization and spontaneous contractility in fibroblasts and suggest that none of these four factors may be dispensable for directly reprogramming fibroblasts to contractile iCMs.

Previous studies quantified the phenotypic effects of reprogramming factors in whole cell population without considering which factors are indeed expressed in individual cells. This blind analysis made it impossible to precisely determine how efficient the reprogramming process is and how many factors are really necessary and sufficient for this process. By visualizing the cells expressing different combinations of reprogramming factors, we demonstrated that at least three factors (GMT) are necessary to induce sarcomeric proteins in most of the transduced cells and that Hand2 expression in the context of GMT expression markedly enhances structural and functional development of iCMs. Our unbiased imaging-based analyses provide new perspectives on the mechanistic basis of inefficient iCM generation and highlights the importance of ensuring expression of four core cardiogenic transcription factors in individual fibroblasts for contractile iCM reprogramming. However, it is important to emphasize that a majority of GHMT expressing fibroblasts still fail to develop contractile structures or to specify a ventricular subtype. Our results indicate that sarcomere assembly and subtype specification of iCMs still remain unresolved challenges for cardiac reprogramming. Taken together, resolving upfront an initial bottleneck for cardiac reprogramming by ensuring expression of all the necessary factors may maximize the effects of additional reprogramming boosters on the subsequent bottlenecks for cardiac reprogramming (i.e. sarcomere organization and subtype specification).

## Methods

### Animals

All animal procedures were performed with the approval of Vanderbilt University Medical Center. All methods were performed in accordance with the relevant guidelines and regulations. Titin-eGFP reporter knock-in mice were kindly provided by Dr. Gotthardt^21^.

### Plasmids

Generation of retroviral vectors encoding mouse Gata4, Hand2, Mef2c, and Tbx5 was described previously^7^. To generate reprogramming factor-fluorescent reporter fusion constructs (i.e. Gata4-eGFP, Hand2-mOrange, Mef2c-tagBFP, and Tbx5-mCherry), PCR amplified cDNAs of each transcription factor and a respective fluorescent reporter are assembled into pBabe-X retroviral vector using NEBuilder HiFi DNA Assembly kit (BioLabs) as per manufacturer’s protocol. To generate tri-cistronic vectors harboring two reprogramming factors and a fluorescent reporter (i.e. M-T-mCherry, M-T-eGFP, M-G-mCherry, M-H-mCherry, mCherry-T-H, mCherry-G-T, mCherry-G-H, tagBFP-G-H), M-*P2A*-T-*P2A*-G or M-*P2A*-G-*P2A*-T tri-cistronic constructs were generated first using pGEMT-easy vector harboring M-*P2A*-T-*T2A*-G or M-*P2A*-G-*T2A*-T^27^ provided by Dr. Qian as a template. For M-*P2A*-T-*P2A*-G construct, Tbx5 in pGEMT-M-*P2A*-T-*T2A*-G was first replaced by PCR amplified myc-tagged Tbx5 cDNA. Then, PCR amplified P2A peptide plus Gata4 cDNA replaced T2A-G in pGEMT-M-*P2A*-myc-tagged T-*T2A*-G upon *T2A*-G excision. M-*P2A*-myc-tagged T-*P2A*-G cassette was excised from the pGEMT vector and subcloned into pBabe-X and pMXs retroviral vector. For M-*P2A*-G-*T2A*-T construct, PCR amplified *P2A*-T replaced *T2A*-T upon *T2A-*T excision in the pGEMT vector. Then, M-*P2A*-G-*P2A-*myc tagged T cassette was subcloned into pMXs retroviral vector. For M-T-mCherry construct, Gata4 was excised and replaced with mCherry in pGEMT-M*-P2A-*myc-tagged T*-P2A-*G and then M-*P2A*-myc-tagged T-*P2A*-mCherry cassette was subcloned into pBabe-X retroviral vector. M-T-eGFP construct was generated by replacing mCherry with eGFP. Similarly, M-G-mCherry construct was generated by replacing Tbx5 in M-*P2A*-G-*P2A*-T pGEMT construct with mCherry and then M-*P2A*-G-*P2A*-mCherry cassette was subcloned into pBabe-X retroviral vector. mCherry-G-T construct was generated by replacing Mef2c in M-*P2A*-G-*T2A*-T pGEMT construct with mCherry and then mCherry-*P2A*-G-*P2A*-T cassette was subcloned into pBabe-X retroviral vector. M-H-mCherry construct was generated by sequentially replacing Tbx5 and Gata4 in M-*P2A*-T-*P2A*-G pGEMT construct with myc-tagged Hand2 and mCherry, and then M-*P2A*-H-*P2A*-mCherry cassette was subcloned into pBabe-X retroviral vector. mCherry-T-H construct was generated by sequentially replacing Mef2c and Gata4 in M-*P2A*-T-*P2A*-G pGEMT construct with mCherry and myc-tagged Hand2, and then mCherry-*P2A*-T-*P2A*-H cassette was subcloned into pBabe-X retroviral vector. For generating mCherry-G-H or tagBFP-G-H construct, pGEMT-M-*P2A*-G-*P2A*-myc-tagged T construct was used as a template. Mef2c and myc-tagged Tbx5 were sequentially replaced by mCherry or tagBFP and Hand2 in pGEMT vector and mCherry or tagBFP-*P2A*-G-*P2A*-H cassette was subcloned into pBabe-X retroviral vector. For pBabe-G-*P2A*-tagBFP, PCR amplified Gata4 and *P2A*-tagBFP cDNAs were directly assembled into pBabe-X vector.

### Isolation of mouse embryonic fibroblasts

E13.5 or E14.5 mouse embryos were harvested from Titin-eGFP reporter^21^ or C57BL/6 (Jackson Laboratory) pregnant mice. The embryos were dissected from the uterus and surrounding membranes. The head, extremities, and the internal organs of the chest and abdominal cavities were completely removed from the embryos. The remaining tissues were minced and digested with 0.25% trypsin for 15 min at 37 °C water bath. The isolated cells were filtered through a cell strainer and plated in 15 cm culture dishes with fibroblast growth medium containing 10% FBS and 1% penicillin/streptomycin. These cells were harvested when cell confluency reaches at around 70–80%.

### Generation of retroviruses

Retroviruses were generated as described previously^7,22^ with minor modifications. Platinum E cells (Cell Biolabs) were transfected at 60–70% confluence with pBabe-X or pMXs retroviral construct DNA using Fugene 6 (Promega). After 24hrs, the viral medium was collected using a syringe and filtered through a 0.45 µm polyethersulfone (PES) membrane filter. Polybrene was added to filtered viral medium at a concentration of 6 µg/ml. The mixture of viral medium and polybrene replaced the fibroblast growth medium in the cell culture dish with MEFs which were plated 16–20 hours earlier. Platinum E cells were replenished with the growth medium (DMEM with 10% FBS and 1% penicillin/streptomycin). Twenty-four hours after first infection, viral medium was sucked off from the cell culture dish and MEFs were re-infected with the second viral medium from Platinum E cell plates. Another 24 hours later, viral medium on the cell culture dish with MEFs was replaced with cardiac induction medium, composed of DMEM/199 (4:1), 10% FBS, 5% horse serum, 1% penicillin/streptomycin, 1% non-essential amino acids, 1% essential amino acids, 1% B-27, 1% insulin-selenium-transferrin, 1% vitamin mixture, and 1% sodium pyruvate (Invitrogen), 1 µM SB431542 (Sigma), and 0.5 µm A83-01 (Tocris). The induction medium was changed every 3 days until cells were harvested.

### Immunocytochemistry

Cells were fixed with 2% paraformaldehyde in cell growth medium for 15 min and permeabilized with permeabilization buffer (0.05% Triton-X in PBS) for 5 min three times at room temperature. Cells were blocked with blocking buffer (Universal blocking buffer, BiogeneX) for 45 min and then incubated with multiple combinations of primary antibodies including anti-α-actinin (Mouse monoclonal, Sigma, 1:400 dilution), anti-GFP (Chicken IgY fraction, Invitrogen, 1:400 dilution), anti-tagRFP (Rabbit polyclonal, Evrogen, 1:400 dilution, for detecting tagBFP), and anti-mCherry (Rat monoclonal, Thermo Fisher Scientific, 1:1000 dilution) for 1.5 hours at room temperature. Following washing three times for 5 min with permeabilization buffer, cells were incubated with appropriate Alexa fluorogenic secondary antibodies (Invitrogen) at 1:400 dilution and DRAQ5 for nuclear staining (Abcam) at 1:500 dilution at room temperature for 1 hour. After another set of washing (5 min x3 with permeabilization buffer), cell images were captured with Zeiss LSM 500 confocal microscope or Olympus IX81 epifluorescent microscope. Alternatively, Hoechst (Invitrogen) staining was used to visualize cellular nuclei. For Hoechst nuclear staining, Hoechst solution was added in washing buffer at final concentration of 2 µM for 15 min after first washing following secondary antibody incubation.

### High content imaging analysis

For high content imaging analysis, cells were seeded at ~10,000 cells per well in 24 well black clear bottom plates (Greiner). Cells were infected and stained directly on 24 well plates as described above. Each 24 well plate included a control well which was not infected, but immunostained with primary and secondary antibodies. After immunostaining, cells were imaged with ImageXpress Micro XL Automated Cell Imaging system (Molecular Device). Images were acquired with a 10X objective at 36 fields per well. DAPI, Texas Red, FITC, and Cy5 filter sets were used to detect tagBFP or Hoechst, mCherry, Titin-eGFP or α-actinin, and DRAQ5 or α-actinin, respectively. Exposure time was determined by autoexposure for nuclear staining. Exposure times for other fluorescent filter sets were manually adjusted to allow minimum positive cells in the control well containing immunostained uninfected cells. Optimal exposure times were determined in each 24 well plate. Captured images were analyzed with MetaXpress software (Molecular Device) to determine the total number of cells by nuclear count and the number of single, double or triple fluorescent positive cells among nuclear stained cells. Some images that contained autofluorescent artifacts (e.g. bubble) were excluded for analysis. Typically, greater than 10,000 cells were counted at 20-36 captured images by nuclear staining in each condition.

### Flow cytometry

MEFs were infected with different combinations of retroviruses expressing a fluorescent reporter-tagged reprogramming factors (i.e. Gata4-eGFP, Hand2-mOrange, Mef2c-tagBFP, and Tbx5-mCherry). Four days after transduction, cells were washed with PBS, detached from the culture dish with 0.125% trypsin, and collected using PBS with 5% FBS. Cells were pelleted by centrifugation at 1,800 g, and re-suspended with stain buffer (BD Bioscience). Then, cells were analyzed for expression of fluorescent reporters using FACS Caliber (BD sciences) and FlowJo software.

### Western blotting

After washing with PBS, cells were harvested with RIPA buffer (Sigma) supplemented with a protease inhibitor tablet (Roche), and then incubated on ice for 30 min. Cells were pelleted by centrifugation at 14,000 rpm for 15 min at 4 °C and cellular lysate was obtained as a supernatant. The cell lysate with SDS loading buffer (Bio-Rad) was boiled for 5 min, and then loaded into 10% or 4%-20% gradient SDS-PAGE gel. Proteins on the gel were transferred into PVDF membranes (Bio-Rad) by electrophoresis. After incubating the membranes in 5% milk blocking solution for 1 hour, the membranes were incubated with a primary antibody prepared in 5% milk solution at room temperature. The primary antibodies used were anti-Gata4 antibody (Rabbit polyclonal, Thermo Scientific), anti-Hand2 antibody (mouse monoclonal, Sigma), anti-Mef2c antibody (Rabbit polyclonal, GeneTex), and anti-Tbx5 antibody (Rabbit polyclonal, Proteintech). Following washing with 0.1% PBS tween-20 three times, the membranes were incubated with a secondary antibody (goat anti-mouse IgG-HRP or goat anti-rabbit IgG-HRP antibody (Bio-Rad)) diluted at 1:5000 in 5% milk solution. Following washing, the membranes were developed using enhanced chemiluminescence substrate (PerkinElmer). The membranes were stripped with stripping buffer (100 mM β-Mercaptoethanol, 2% SDS, 62.5 mM Tris-HCL, pH 6.8 in 0.1% PBS tween-20) and re-probed with mouse anti-GAPDH antibody (Santa Cruz Biotechnology) for loading control.

## Supplementary information

Supplementary Figures and Figure Legends

Online Movie 1

Online Movie 2

Online Movie 3

Online Movie 4

Online Movie 5

Online Movie 6

Online Movie 7

Online Movie 8

## Data Availability

The datasets generated during and/or analysed during the current study are available from the corresponding author on reasonable request.
