# Supplementary material for: Ensuring expression of four core cardiogenic transcription factors enhances cardiac reprogramming
Source: Sci Rep. 2019 Apr 24;9:6362. doi: 10.1038/s41598-019-42945-w (PMC6482135; doi:10.1038/s41598-019-42945-w)
Supplement: Supplementary file 1 — Supplementary Figures and Figure Legends [file 41598_2019_42945_MOESM1_ESM.pdf]

SUPPLEMENTARY FIGURES and FIGURE LEGENDS

Title: Ensuring expression of four core cardiogenic transcription factors enhances cardiac reprogramming

Zhentao Zhang[1, 2, 3], Alexander D. Zhang[1, 2, 3], Luke J. Kim[1, 2, 3], and Young-Jae Nam[1, 2, 3, *]

[1]Department of Medicine, Division of Cardiovascular Medicine, Vanderbilt University Medical Center, Nashville, TN, USA
[2]Department of Cell and Developmental Biology, Vanderbilt University, Nashville, TN, USA
[3]Vanderbilt Center for Stem Cell Biology, Vanderbilt University, Nashville, TN, USA

*To whom correspondence may be addressed:
Young-Jae Nam, M.D., Ph.D.
Department of Medicine, Division of Cardiovascular Medicine
Vanderbilt University Medical Center
Nashville , Tennessee 37232, USA
Phone: 615-936-5436
E-mail: young-jae.nam@vanderbilt.edu

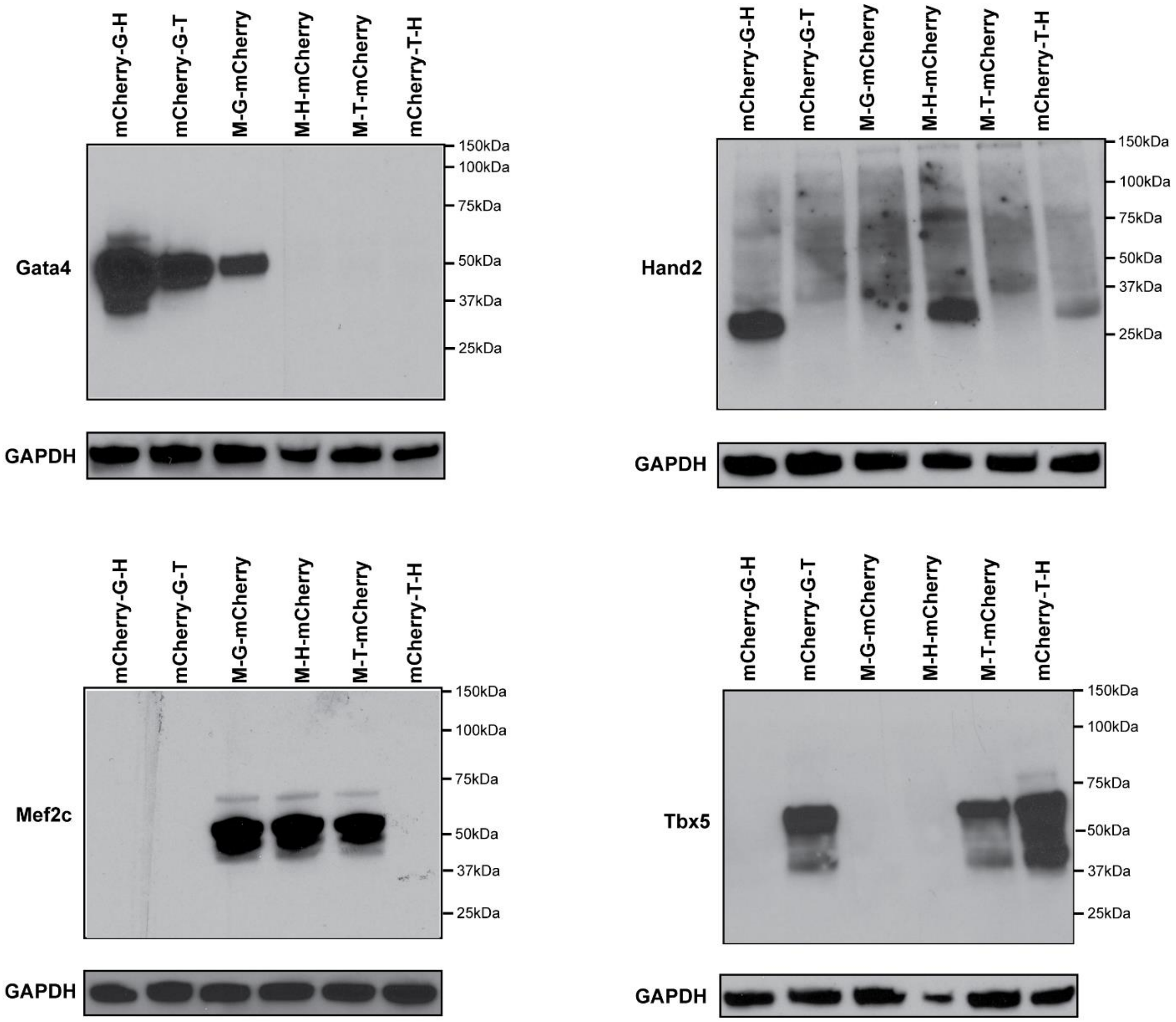

Figure S1. Protein expression of cardiogenic transcription factors harbored in tri-cistronic retroviral vectors. MEFs were transduced with retroviruses encoding indicated transcription factors with mCherry. Using the lysates from the transduced MEFs, protein expression of indicated transcription factors was detected by western blot with anti-Gata4, anti-Hand2, anti-Mef2c, and anti-Tbx5 antibody.

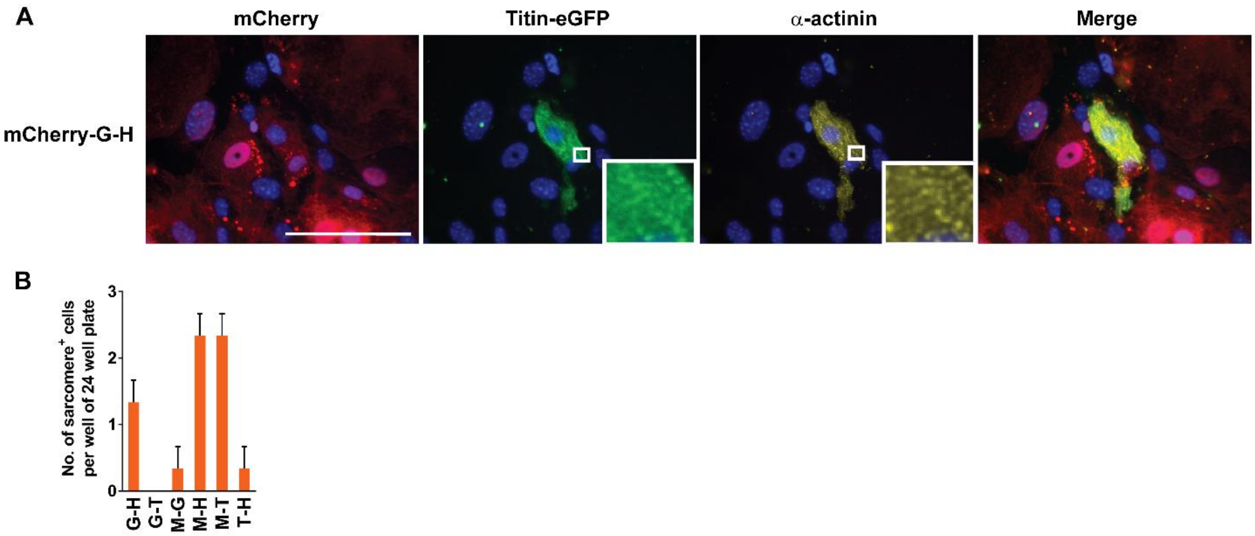

Figure S2. Sarcomere organization by forced expression of two cardiogenic transcription factors. (A) A representative immunofluorescent image demonstrating organized sarcomeric structures in cells expressing two factors. White boxes were enlarged in insets to demonstrate organized M-band (Titin-eGFP) and Z-band (α-actinin). Scale bar, 100 µM. (B) Quantification of well-organized sarcomere$^+$ cells identified by visualizing M-band structures with Titin-eGFP expression after transduction of indicated two factors. Three independent experiments are presented as mean$\pm$s.d.

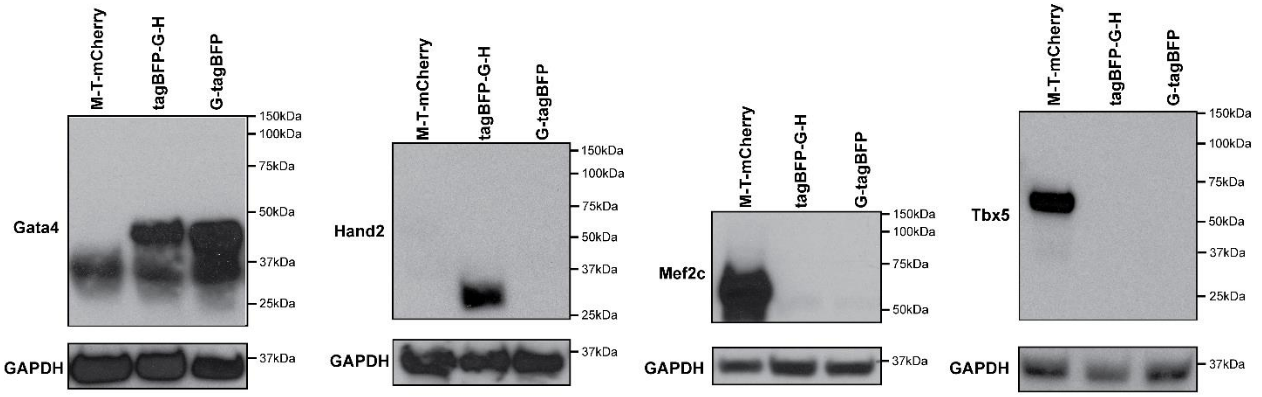

Figure S3. Protein expression of cardiogenic transcription factors harbored in tri- or bi-cistronic retroviral vectors. MEFs were transduced with retroviruses encoding indicated transcription factors with mCherry or tagBFP. Using the lysates from the transduced MEFs, protein expression of indicated transcription factors was detected by western blot with anti-Gata4, anti-Hand2, anti-Mef2c, and anti-Tbx5 antibodies. For Mef2c blotting, the membrane was cut between 37 kDa and 50kDa after protein transfer, and each membrane was separately blotted for Mef2c and GAPDH.

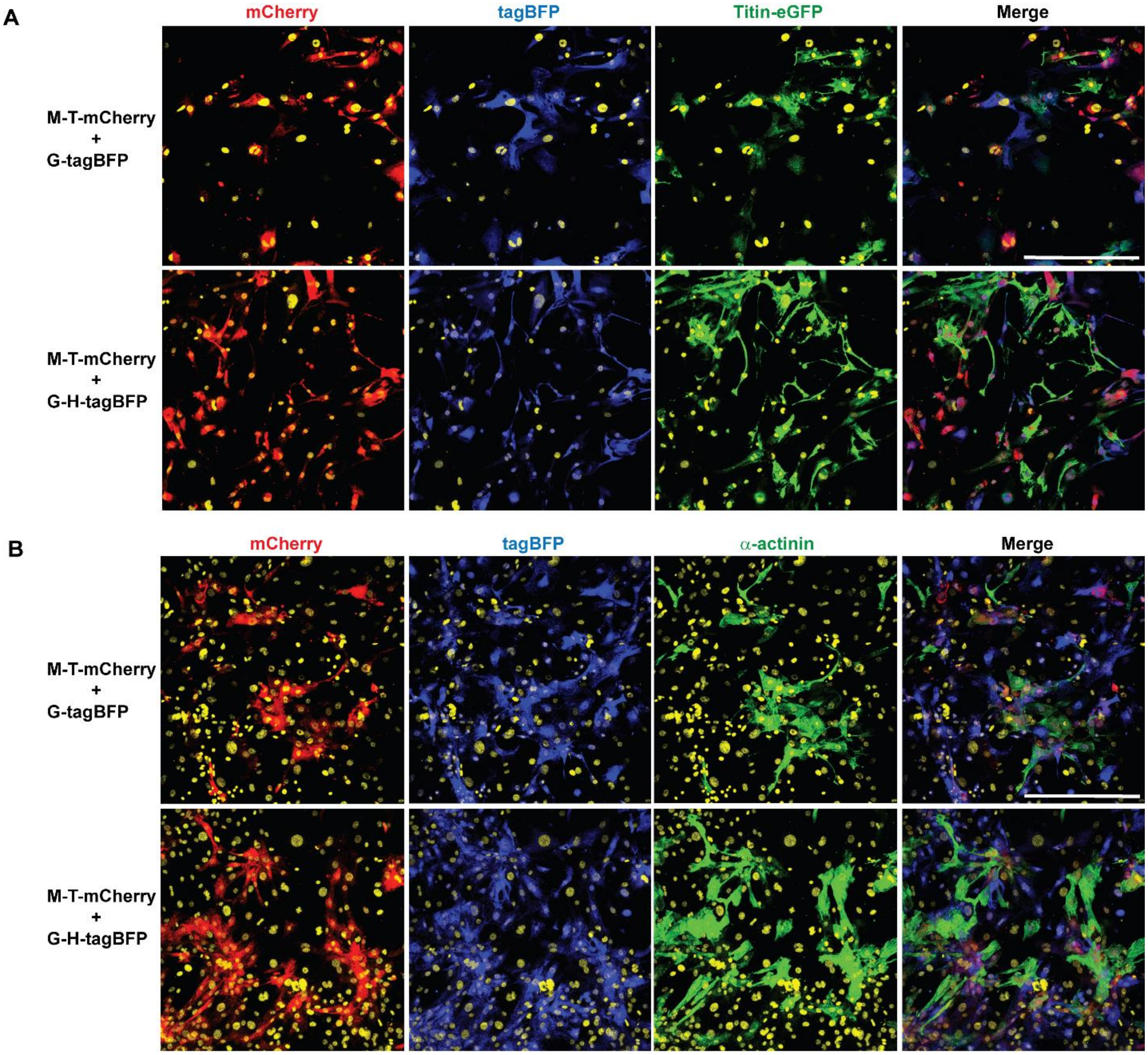

Figure S4. **Visualization of sarcomere protein induction in GMT or GHMT expressing cells.** Representative immunofluorescent images of the cell analyzed by the high content imaging system in Figure 2 were shown. Simultaneous expression of Titin-eGFP (A) or α-actinin (B) and mCherry and tagBFP was visualized using 10X objective. mCherry expression indicates expression of Mef2c and Tbx5. tagBFP expression indicates expression of Gata4 (upper panel) or Gata4 and Hand2 (lower panel). Nuclei are stained with DRAQ5 (yellow). Scale bar, 400 µM.

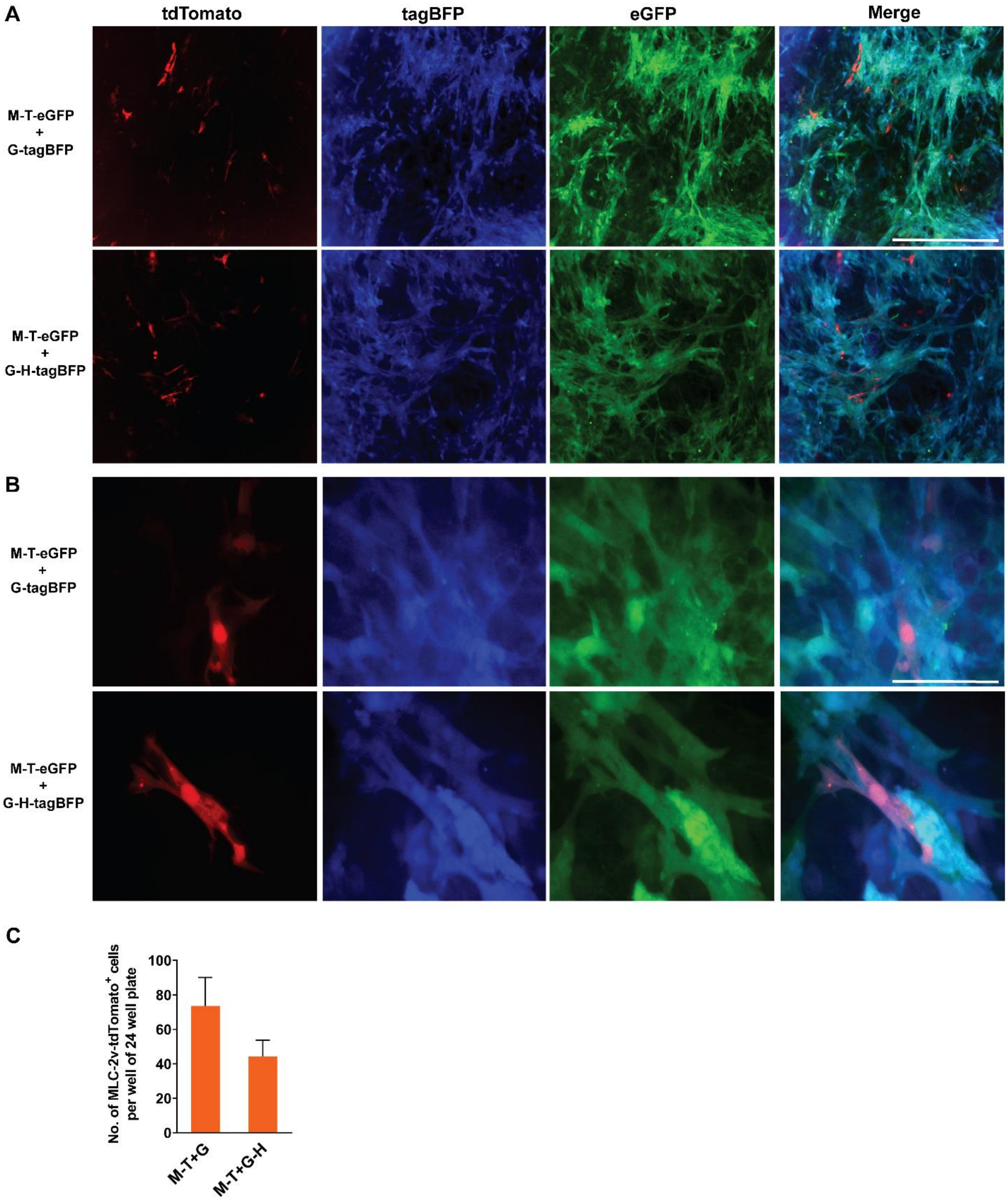

Figure S5. Induction of ventricular specific MLC-2v in GMT or GHMT expressing cells. Representative immunofluorescent images for MLC-2v-tdTomato expressing cells following M-T-eGFP and G-tagBFP or G-H-tagBFP transduction using a 10X (A) or 40X (B) objective. Nearly all tdTomato expressing cells express eGFP and tagBFP. The indicated combinations of vectors were transduced into MEFs isolated from MLC-2v-tdTomato reporter knock-in mice. Immunofluorescence staining was performed at day 15 post-transduction. Scale bar, 400 μM

(A) and 100 µM (B). (C) Summary of quantification of MLC-2v-tdTomato$^+$ cells among GMT or GHMT expressing cells. tdTomato$^+$eGFP$^+$tagBFP$^+$ cells were manually counted under an epifluorescence microscope using a 20X objective. Eleven independent experiments are presented as mean$\pm$s.d. *P*>0.1.

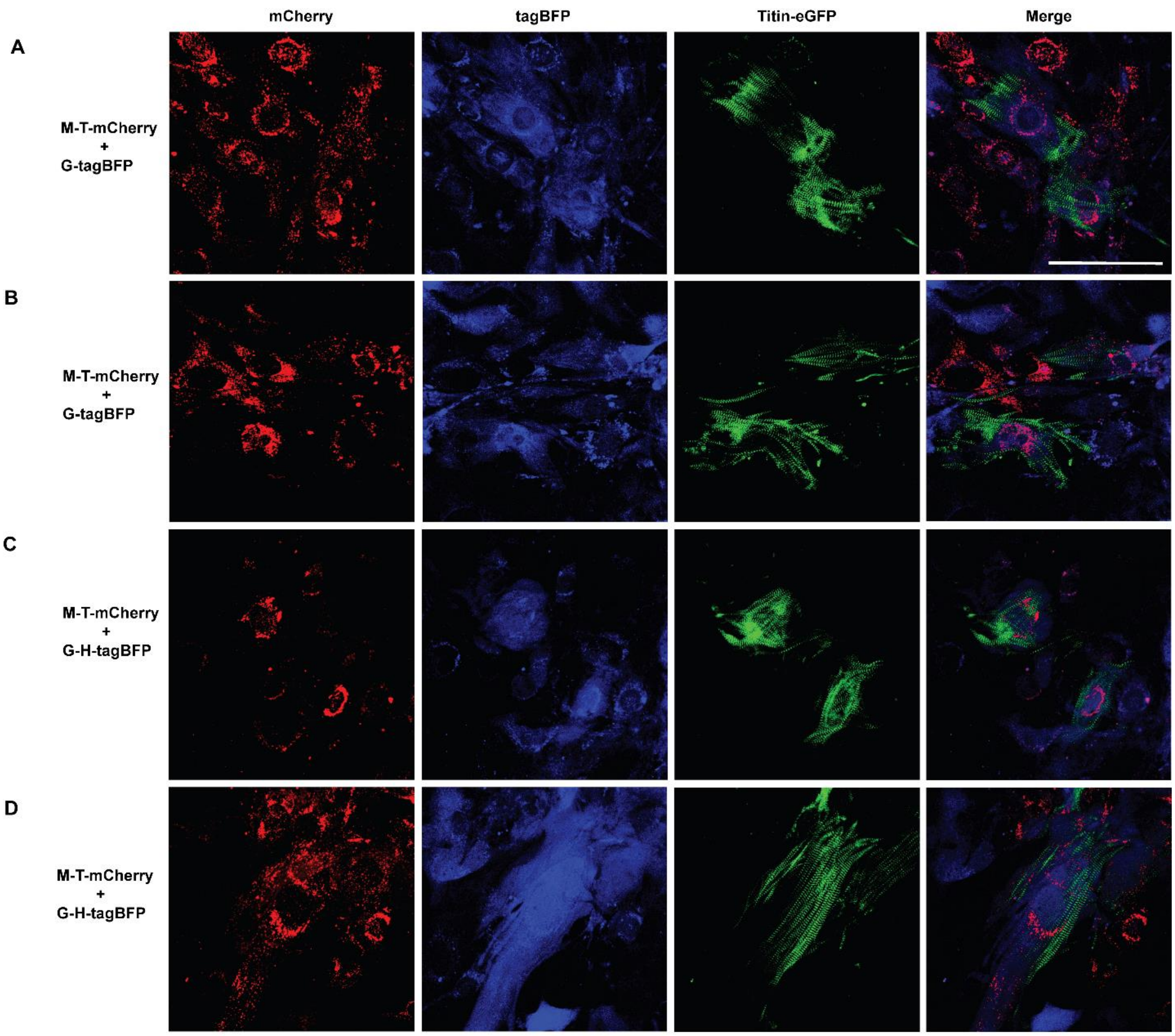

Figure S6. Simultaneous visualization of both sarcomeric structures and GMT or GHMT expression in contractile iCMs. Immunofluorescent images were taken without immunostaining in spontaneously beating iCMs derived from Titin-eGFP reporter MEFs following transduction of M-T-mCherry and G-tagBFP or G-H-tagBFP. The movies for spontaneous contraction of these cells are shown in Online Movies 5-8 (A: Online Movie 5, B: Online Movie 6, C: Online Movie 7, and D: Online Movie 8). Scale bar, 100 μM.

Online Movie 1-4. Spontaneously calcium oscillation visualized by GCaMP3 reporter. Two to three weeks after transduction of MEFs isolated from αMHC-Cre: Rosa26-GCaMP3 mice with GMT (Online Movie 1 and 2) or GHMT (Online Movie 3 and 4), GCaMP3 reporter expression was visualized.

Online Movie 5-8. Spontaneously beating of iCMs. Two to three weeks after transduction of MEFs isolated from Titin-eGFP reporter knock-in mice with GMT (Online Movie 5 and 6) or GHMT (Online Movie 7 and 8), spontaneous contraction of M-band structures is visualized.
